# MR-guided stereotactic body radiation therapy for primary cardiac sarcomas

**DOI:** 10.1186/s13014-021-01791-9

**Published:** 2021-03-26

**Authors:** Stefanie Corradini, Rieke von Bestenbostel, Angela Romano, Adrian Curta, Dorit Di Gioia, Lorenzo Placidi, Maximilian Niyazi, Luca Boldrini

**Affiliations:** 1 Department of Radiation Oncology, University Hospital, LMU Munich, Munich, Germany; 2 Department of Bioimaging, Radiation Oncology and Hematology, Fondazione Policlinico Universitario “A. Gemelli” IRCCS, Rome, Italy; 3 Department of Radiology, University Hospital, LMU Munich, Munich, Germany; 4 Department of Medicine III, University Hospital, LMU Munich, Munich, Germany

**Keywords:** Magnetic resonance guided radiation therapy, Primary cardiac sarcoma, Magnetic resonance stereotactic body radiation therapy

## Abstract

**Background:**

Primary cardiac tumors are an extremely rare disease with limited prognosis. The treatment of choice is surgery. Other treatment options include chemotherapy and radiation therapy, which historically represented a palliative approach in patients who were not eligible for surgery. The development of hybrid MR-guided radiation therapy makes it possible to better visualize cardiac lesions and to apply high doses per fraction in sensible organs such as the heart.

**Case presentation:**

Patients affected by inoperable primary cardiac sarcomas and treated at two different institutions were considered for this analysis and retrospectively analyzed. All patients were treated using a 0.35 T hybrid MR Linac system (MRIdian, ViewRay Inc., Mountain View, CA). In the present study we investigated the feasibility, early outcome and toxicity of MR-guided RT in primary cardiac sarcomas. Four consecutive non-metastasized patients who were treated between 05–09/2020 were analyzed. The cardiac sarcomas were mostly located in the right atrium (50%) and one patient presented with 3 epicardial lesions. All patients received MRgRT as a salvage treatment for recurrent cardiac sarcoma after initial surgery, after a mean interval of 12 months (range 1–29 months). Regarding the treatment characteristics, the mean GTV size was 22.9 cc (range 2.5–56.9 cc) and patients were treated with a mean GTV dose of 38.9 Gy (range 30.1–41.1 Gy) in 5 fractions. Regarding feasibility, all treatments were completed as planned and all patients tolerated the treatment very well and showed only mild grade 1 or 2 symptoms like fatigue, dyspnea or mild chest pain at early follow-up.

**Conclusion:**

To the best of our knowledge, in this retrospective analysis we present the first and largest series of patients presenting with primary cardiac sarcomas treated with online adaptive MRgRT. However, further studies are needed to evaluate the impact of this new methodology on the outcome of this very rare disease.

**Supplementary Information:**

The online version contains supplementary material available at 10.1186/s13014-021-01791-9.

## Background

Primary cardiac tumors are an extremely rare disease with a reported incidence of 0.001–0.28% in autopsy series and represent a heterogeneous group of neoplasms with different clinical courses and histologies. The vast majority of tumors are benign and only 25% of cases have malignant histology. Sarcomas account for 75–95% of malignant presentations, with primary angiosarcomas accounting for 20–30% of histological subtypes [1, 2].

As far as treatment options are concerned, there are no uniform guidelines for the management of cardiac sarcomas due to disease rarity [3]. If feasible, the treatment of choice is surgery. However, in many cases the clinical presentation does not allow radical surgical excision, which limits the impact on prognosis, which remains extremely poor [3, 4]. Recently, heart transplant has also been introduced as an emerging strategy for patients with isolated inoperable cardiac involvement [3, 5]. Other treatment options include chemotherapy and radiation therapy, which historically represented a palliative approach in patients who were not eligible for surgery, or as part of multimodal treatment strategies [2, 3].

Nevertheless, the latest technological developments in the field of radiation oncology may expand the current treatment options, leading towards clinical outcome improvement. The introduction of real-time magnetic resonance image (MRI)-guided radiotherapy (MRgRT) represents one of the most innovative applications of modern image-guided radiation therapy, as it enables a direct visualization of the target—even during treatment delivery itself [6]. Novel hybrid devices combine magnetic resonance imaging and a linear accelerator in a single device. The revolutionary concept of MRgRT is the use of high-quality image guidance, online plan adaptation workflows and automated therapy volumes gating capabilities.

In the specific case, MRgRT makes it possible to better visualize the cardiac lesions thanks to the improved soft tissue contrast of MRI and to effectively manage respiratory organ motion by means of automatic gating solutions with online MR imaging during dose delivery [7, 8]. Furthermore, promising experiences showed the feasibility of cardiac MRgRT also for non-oncological diseases in cases of refractory ventricular tachycardia and support the use of such advanced delivery techniques for cardiac irradiation [9]. Here, we report a series of patients with primary cardiac sarcomas treated with MR-guided stereotactic body radiation therapy.

## Case presentation

Patients affected by inoperable primary cardiac sarcomas and treated at two different institutions at the University Hospital LMU Munich (Germany) and Fondazione Policlinico Universitario “A. Gemelli” IRCCS of Rome (Italy) were considered for this analysis and retrospectively analyzed. General selection criteria were histologic diagnosis of a primary non-metastatic cardiac sarcoma, age > 18 years, Eastern Cooperative Oncology Group (ECOG) status ≤ 3.

In all patients, the presence of cardiac sarcoma was confirmed histologically via prior surgery. Moreover, all patients underwent a complete cardiologic examination to assess cardiac function (transthoracic or transesophageal echocardiography, echocardiogram) and diagnostic imaging using cardiac MRI or CT. The treatment was conducted after the indication has been given and approved by an interdisciplinary tumor board to confirm the most appropriate therapeutic option. All patients were treated using a 0.35 T hybrid MR Linac system (MRIdian, ViewRay Inc., Mountain View, CA). Before treatment initiation, all patients were screened for MRI compatibility and instructed in MRI safety procedures.

Regarding treatment planning, all patients underwent a MR simulation on the 0.35 T MRIdian system using a balanced steady-state free progression (TrueFISP) imaging sequence. The patients were positioned with both arms elevated above the head using a patient positioning device. Various sequences were acquired to obtain a reproducible and stable breath-hold. Thereafter, a planning CT using the same patient positioning and the same breath-hold was performed to acquire the electron density information for treatment planning. The two imaging-sets were fused and target delineation was performed. The target volume (GTV) and organs at risk (heart, heart-valves, heart-minus-PTV, aorta, spinal cord, spinal canal, lungs, trachea, esophagus) were delineated on the MRI. The GTV was then isotropically expanded by 3–5 mm to define the PTV.

The dose prescription was in the range of 5 × 6–7 Gy prescribed at the mean or 80% isodose, depending on the tumor size and localization of the sarcoma and was individualized on a case-by-case basis. The planning constraints applied to OARs are summarized in Table 1. Treatment planning was carried out using the dedicated MRIdian treatment planning software (MC dose calculation algorithm) and consisted of a step and shoot IMRT technique [10]. The linear accelerator (linac) consists of a 6 MV flattening filter free and overall 10–13 beams were used with 40–90 segments.

**Table 1:** Dose constraints to OARs for treatment planning in cardiac sarcoma

Structure	Volume to dose	Dmax (Gy)
Spinal cord	< 0.35 cc at 23 Gy	30
Heart valves (aortic, pulmonary, mitral, tricuspid) *excluding PTV*	< 0.5 cc at 23 Gy *soft constraint	38
Left ventricle *excluding PTV*	< 1 cc at 36 Gy	38
Heart *excluding PTV*	< 15 cc at 32 Gy	38
Great vessels	< 10 cc at 47 Gy	53
Trachea	< 4 cc at 16.5 Gy	40
Bronchus	< 0.5 cc at 21 Gy	33
Esophagus	< 1 cc at 19.5 Gy	35
Bowel	< 0.5 cc at 33 Gy	
Stomach	< 0.5 cc at 33 Gy	

All patients were treated using an online adaptive workflow, where a new treatment plan was optimized after online re-contouring of the anatomy of the day if deemed necessary. During dose delivery, continuous real-time 2D-cine-MRI was used to control for tumor motion. For this purpose, repeated fast planar cine-MRI in a sagittal plane with four frames per second were used for intra-fractional motion monitoring and gating [10]. The system can automatically gate the beam by using real-time anatomy structure tracking [11].

For this purpose, a target structure (cardiac GTV) was defined in the sagittal view of the volumetric MRI, and a surrounding gating boundary contour was created by adding an appropriate tracking margin of 3–5 mm. The tracking algorithm deforms the anatomical contour on every subsequent live cine MRI frame and compares it to the static boundary contour. If the anatomy of interest moves outside the boundary, the beam is automatically stopped until the tracking volume returns into the boundary. The percentage of the target that may be outside the boundary before the beam is shut-off was adjusted to 3% (see Additional file 1: Video). This eliminates the need of the application of an internal target volume (ITV) in order to account for intrafractional respiratory-related target motion [12].

In the present study we evaluated the feasibility and early outcome of MR-guided RT for primary cardiac sarcomas. Toxicity was reported according to Common Terminology Criteria for Adverse Events (CTCAE) v5.

Overall, we identified four consecutive patients who were included in the present study and were treated between 05–09/2020. Patients characteristics are listed in Table 2. Three patients were female and the median age was 64 years (range 49–80 years). The cardiac sarcomas were mostly located in the right atrium (50%) and one patient presented with 3 epicardial lesions. None of the patients had evidence of metastatic disease of the cardiac sarcoma at the time of RT, however, one of the patients had also a history of metastasized melanoma, which was stable at the time of cardiac RT.

**Table 2:** Patient characteristics

	Patient 1 (LMU)	Patient 2 (LMU)	Patient 3 (LMU)	Patient 4 (A. Gemelli)
Histology	Fibroblastic/myofibroblastic sarcoma, G1	Pleomorphic cardiac sarcoma, G3	Biphasic synovial sarcoma, G2	Pleomorphic cardiac sarcoma, G3
Localization	Left atrium	Right atrium	Epicardial right (3 lesions)	Right atrium/ventricle
Age at time of treatment (years)	49	80	61	66
Gender	Female	Female	Female	Male
Karnofsky score (KPS)	90	60	80	60
Symptoms before treatment	Fatigue CTCAE °1	Fatigue CTCAE °1 dyspnea CTCAE °1	Fatigue CTCAE °1 chest pain CTCAE °1	Fatigue CTCAE °1 dyspnea CTCAE °2
Prior surgery	Yes; R1	Yes; R2	Yes, R0 (close)	Yes, R2
Time interval surgery to RT (months)	7	1	29	11
Chemotherapy	No	No	Yes, neoadjuvant 3 courses doxorubicin/ifosfamide 75/10 and 1 course adjuvant	Yes, adjuvant 6 courses epirubicin/ifosfamide

All patients received MRgRT as a salvage treatment for recurrent cardiac sarcoma after initial surgery, after a mean interval of 12 months. Two patients with high grade sarcoma also received chemotherapy before undergoing RT. Regarding the symptoms prior to RT, most patients had only mild symptoms with mainly fatigue (CTCAE°1), shortness of breath (CTCAE°1), or mild chest pain (CTCAE°1).

**Fig. 1:**
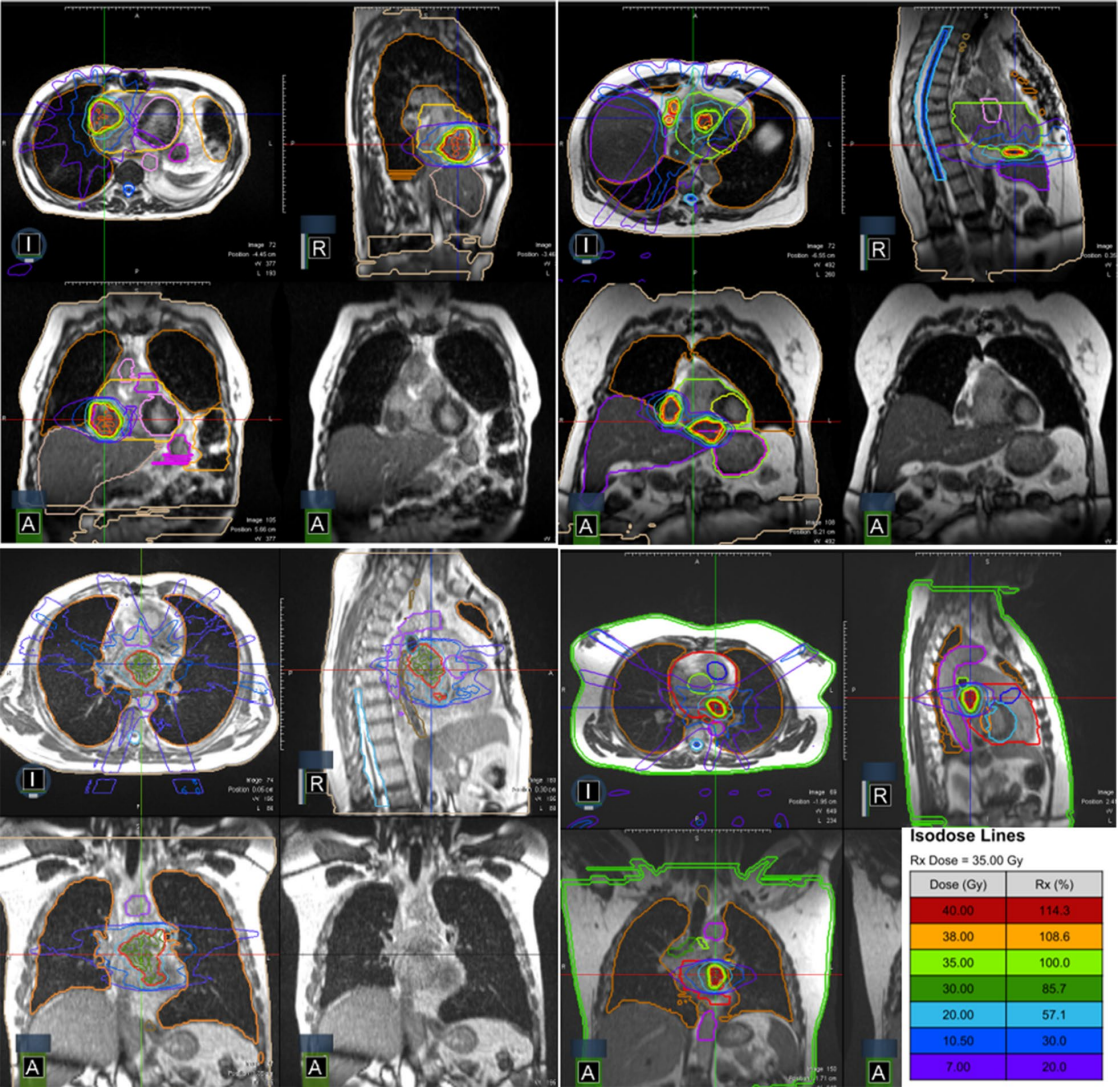
Exemplar MR-guided RT plans for the treatment of primary cardiac sarcoma

Regarding the treatment characteristics, the mean GTV size was 22.9 cc (range 2.5–56.9 cc) and the mean PTV volume was 35.5 cc (range 5.8–87.1 cc). The dose prescription was 5 × 7 Gy prescribed to the 80% isodose line in the 3 cases treated at LMU (see Fig. 1) and considering the large dimensions of the lesion treated at A. Gemelli, a total dose of 5 × 6 Gy per fraction was prescribed to the PTV [13]. This resulted in a mean PTV dose of 37.5 Gy (range 29.6–39.9 Gy) and a mean GTV dose of 38.9 Gy (range 30.1–41.1 Gy) in 5 fractions. The heart volume (excluding the PTV) received a mean dose of 6.6 Gy (range 3.5–8.5 Gy). The details are reported in Table 3. In Fig. 1 are shown exemplar MR-guided RT plans for the treatment of primary cardiac sarcoma.

**Table 3:** Treatment characteristics

	Patient 1 (LMU)	Patient 2 (LMU)	Patient 3 (LMU)			Patient 4 (A. Gemelli)
Site of RT	Left atrium	Right atrium	Epicardial right			Right atrium/ventricle
Number of lesions	1	1	3			1
Pre-treatment Size (cm^3^)	2.3 × 1.3 × 2.1	4.6 × 4.0 × 4.3	2.8 × 3.2 × 1.5	4.7 × 4.4 × 3.0	1.6 × 1.2 × 1.9	5.6 × 5.8 × 7.2
Prescription total dose (Gy)	35	35	35			30
Prescription single dose (Gy)	7	7	7			6
Isodose prescription	80%	80%	80%			Dmean
Treatment on	Non-consecutive days	Non-consecutive days	Non-consecutive days			Daily
Lesion	GTV1	GTV1	GTV1	GTV2	GTV3	GTV1
GTV volume (cc)	3.13	38.02	12.94	24.03	2.51	56.9
GTV Dmin (Gy)	37.29	36.14	36.71	29.58	36.56	12.9
GTV D98% (Gy)	38.52	38.08	38.85	37.80	38.68	25.52
GTV Dmax (Gy)	41	43.27	43.54	44.47	43.37	33.13
GTV D2% (Gy)	40.81	42.53	42.86	43.27	42.98	31.84
GTV Dmean (Gy)	39.72	40.78	41.12	41.03	40.92	30.06
PTV	PTV1	PTV1	PTV1	PTV2	PTV3	PTV1
PTV volume (cc)	8.99	55.39	17.71	38.12	5.78	87.12
PTV Dmin (Gy)	31.83	25.97	30.77	23.92	31.62	10.74
PTV D98% (Gy)	34.62	29.24	35.01	34.05	35.14	23.14
PTV Dmax (Gy)	41	43.27	43.54	44.47	43.37	33.13
PTV D2% (Gy)	40.56	42.32	42.73	43.02	42.76	31.75
PTV Dmean (Gy)	38.29	38.03	39.88	39.92	39.5	29.61
Heart sub PTV Dmax (Gy)	37.66	35.4	38.44			31.12
Heart sub PTV Dmean (Gy)	3.54	6.33	7.87			8.46
MU	2203	1894	4208			3836
Beams	13	12	10			10
Segments	44	40	79			90
Feasibility (treatment delivered within planned time schedule)	100%	100%	100%			100%

Regarding feasibility, all treatments were completed as planned within 5 (non-)consecutive days and no interruptions were reported. All patients tolerated the treatment very well and showed no acute toxicity at the end of treatment. The median follow-up was 4 months. So far, all lesions were locally stable, with no signs of progression. However, one patient showed a systemic progression with the appearance of distant metastasis and received systemic therapy. Another patient with a history of metastasized melanoma had a systemic progression of the melanoma with the appearance of three secondary lesions (two hepatic and one adrenal), while the cardiac sarcoma remained stable. Overall, toxicity at the first follow-up showed only mild grade 1 or 2 symptoms like fatigue, dyspnea or mild chest pain (see Table 4).

**Table 4:** Follow-up and toxicity

	Patient 1 (LMU)	Patient 2 (LMU)	Patient 3 (LMU)	Patient 4 (A. Gemelli)
Time from last RT to follow up (months)	6	6	3	1
Local control	Yes	Yes	n.a	Yes
Metastases	No	Yes, mediastinal lymphnode metastases, liver metastases	n.a	[Yes, from melanoma]
Toxicity at first follow-up	Fatigue CTCAE °1 Dyspnea CTCAE °1	Fatigue CTCAE °2 Dyspnea CTCAE °2 Chest pain CTCAE °1	None	Fatigue CTCAE °2 Dyspnea CTCAE °2

## Discussion

Primary cardiac sarcomas are very rare and the symptoms are manifold and depend on the location of the tumor. They range from heart-specific symptoms such as cardiac arrhythmias, congestive heart failure, cardiac tamponade, pericardial effusions to general symptoms such as chest pain, shortness of breath, syncope or neoplastic accompanying symptoms like fever, weight loss and malaise [5]. This unspecific symptomatology contributes to the difficulty in diagnosing cardiac sarcomas and many cardiac neoplasms remain undetected during lifetime and are only diagnosed post-mortem. This corresponds to the experience of the present series, where most patients presented with very mild and unspecific symptoms, as fatigue. Often, more specific clinical findings manifest later in the course of the disease until the tumor has increased to a certain size or the patient has developed regional spread or metastases [2].

Therefore, the level of evidence for the optimal multimodal management of primary cardiac sarcomas is low, due to disease rarity. A retrospective analysis of the French Sarcoma Group reviewed 124 cases regarding treatment modalities and outcome of primary sarcomas treated between 1977 and 2010 [3], of which 20% had primary metastasis. This cohort gives a good overview of multimodal treatment strategies in cardiac sarcoma. Overall, 65% of patients underwent surgery and 4% received heart transplant. Radiotherapy was performed in 24 non-metastatic in an adjuvant setting (75%) or as a definitive treatment option (25%). Chemotherapy, on the other hand, was administered very frequently in 90% of all patients. Regarding outcome, there was a significant correlation between the extent of surgery and survival outcome. The median overall survival for the entire cohort was 17 months, while it was 38 months following a complete resection (achieved in only 13%) compared to 18 months after incomplete resection (R1/R2) and only 11 months in patients not receiving surgery at all. Likewise, chemotherapy and radiotherapy had an impact on progression-free survival and overall survival.

In the French series, radiotherapy was presumably delivered using CT-based image guidance with difficulties in managing breathing-related organ motion of the heart-taking into account the available radiotherapy techniques at that time (1977–2010). Therefore, larger target volumes were probably used to account for these uncertainties. Furthermore, in order to prevent a myocardial injury through RT, conventionally fractionated regimens with total doses ranging around 50 Gy were applied [3, 14]. However, the study showed that RT was still associated with better overall survival through the improvement of locoregional control rates, even with the application of relatively small total doses for the definitive treatment of sarcomas. This is where modern RT techniques come into play. As extrapolated from data regarding definitive RT of extra-cardiac soft-tissue sarcomas, local control rates significantly depend from total dose and tumor size [15]. The use of modern RT techniques offers the possibility to fully exploit the therapeutic window by achieving superior dose distributions and thus allow for dose escalation strategies with acceptable toxicity rates [15]. For RT treatments within sensitive OARs like the heart, SBRT has the capability to deliver large doses of radiation in a few fractions and is an established technique in the ablative treatment of metastases. Over the past years, stereotactic radiotherapy has become a new alternative treatment option for functionally inoperable cardiac and pericardiac malignancies. Several case reports and small case series have reported their experiences in the treatment of primary heart sarcomas. An overview is given in Table 5. Furthermore, recently Sim et al. [16] reported their experience on the treatment of cardiac metastases using MR-guided SBRT. The technique is comparable to the one used in this series.

**Table 5:** Overview of available literature

Primary sarcomas							
Publication	Histology	Localization	Number of patients	Dose	Response rate	Survival	Toxicities
Aoka 2004 [1]	Primary cardiac angiosarcoma	Right atrium	1	64 Gy/4 Gy carbon-ion radiotherapy, followed by recombinant Interleukin-2	Locally SD, lung metastases after 4 months	Alive at 1.5 years after treatment	None
Bonomo 2015 [17]	Primary cardiac angiosarcoma and malignant melanoma metastasis	Cardiac lesions	3	3 × 8 Gy (80% ID) 5 × 6 Gy (80% ID)	PR – 2 (PCA) SD – 1 (MM)	Alive at 6 months after treatment, after 8 months 1 patient died (lung metastasis)	None
Gabani 2019 [13]	Primary cardiac angiosarcoma	Right atrium (7.3 cm)	1	5 × 6 Gy daily, concurrent paclitaxel (80 mg/m^2^) weekly	After 6 weeks: ipilimumab/nivolumab → PR: 4.1 cm but progression of pulmonary metastasis	Alive at 6 months after treatment	Esophagitis
Isambert 2013 [3]	Primary cardiac sarcoma	Cardiac lesions	124 of which 24 were treated with radiotherapy: 18 adjuvant and 6 definitive	Median dose 50 Gy (range 10–64 Gy). 23 followed by chemotherapy	Benefit in PFS	mOS 17.2 months (all), better survival with radiotherapy (HR 0.54, inivariate; HR 0.62 multivariate analysis)	n.a.
Nakamura-Horigome 2008 [18]	Primary cardiac angiosarcoma	Right atrium, right ventricular wall	1	42 Gy/2 Gy Simultaneous radiochemotherapy with docetaxel & 12 months low dose docetaxel	SD	Alive at 12 months after treatment	Esophagitis
Pötter 1989 [19]	Primary cardiac angiosarcoma	Right atrium	1	40 Gy/1.8 Gy whole heart/mediastinum, boost with 60 Gy to the right atrium, simultaneous radiochemotherapy	Macroscopic no tumor at autopsy	OS 15 months, died of progressive brain metastases	None
Soltys 2008 [20]	Pulmonary artery intimal sarcoma	Left pulmonary artery	1	3 × 11 Gy (79% ID)	Improvement of dyspnea	Died 10 weeks after SBRT due to metastatic progression	None

Real-time MRgRT allows to acquire high-quality MR images immediately before and in real-time during the treatment itself. Moreover, the superior soft tissue contrast compared to cone-beam computed tomography (CBCT)-based RT, gives the opportunity for daily online plan adaptation strategies to improve target volume coverage while avoiding nearby critical structures [6]. Especially in the case of cardiac malignancies, MRgRT enables a direct visualization of the tumor. Moreover, a tracking algorithm allows to track the lesion automatically in real-time during the treatment and therefore planning volume margins can be kept small. This might be correlated to a limited toxicity, as reported in the early experience of this series.

## Conclusion

To the best of our knowledge, in this retrospective analysis we present the first and largest series of patients presenting with primary cardiac sarcomas treated with online adaptive MRgRT. However, further studies are needed to evaluate the impact of this new methodology on the outcome of this very rare disease. Therefore, the authors plan a prospective multicenter trial to generate more evidence for this approach.

## Supplementary Information

**Additional file 1**. The target (blue) and the corresponding boundary (red). The beam is automatically triggered when the target is positioned in the boundary.

## Data Availability

Not applicable.

## Abbreviations

CT: Computed tomography
CTCAE: Common Terminology Criteria for Adverse Events
GTV: Gross tumor volume
ITV: Internal target volume
MRI: Magnetic resonance imaging
MRgRT: Magnetic resonance-guided radiation therapy
PTV: Planning target volume
RT: Radiation therapy
SBRT: Stereotactic body radiation therapy
